# The independent prospective associations of activity intensity and dietary energy density with adiposity in young adolescents

**DOI:** 10.1017/S0007114515005097

**Published:** 2016-01-13

**Authors:** Esther M. F. van Sluijs, Stephen J. Sharp, Gina L. Ambrosini, Aedin Cassidy, Simon J. Griffin, Ulf Ekelund

**Affiliations:** 1 School of Clinical Medicine, Medical Research Council Epidemiology Unit & UK Clinical Research Collaboration (CRC) Centre for Diet and Activity Research, University of Cambridge, Cambridge CB2 0QQ, UK; 2 Medical Research Council Human Nutrition Research, Cambridge CB1 9NL, UK; 3 School of Population Health, The University of Western Australia, Perth, WA 6009, Australia; 4 Department of Nutrition, Norwich Medical School, University of East Anglia, Norwich NR4 7UQ, UK; 5 Norwegian School of Sport Science, 0806 Oslo, Norway

**Keywords:** Physical activity, Sedentary behaviours, Dietary energy density, Adiposity, Prospective cohort studies, Epidemiology, Children and adolescents

## Abstract

There is limited evidence on the prospective association of time spent in activity intensity (sedentary (SED), moderate (MPA) or vigorous (VPA) physical activity) and dietary intake with adiposity indicators in young people. This study aimed to assess associations between (1) baseline objectively measured activity intensity, dietary energy density (DED) and 4-year change in adiposity and (2) 4-year change in activity intensity/DED and adiposity at follow-up. We conducted cohort analyses including 367 participants (10 years at baseline, 14 years at follow-up) with valid data for objectively measured activity (Actigraph), DED (4-d food diary), anthropometry (waist circumference (WC), %body fat (%BF), fat mass index (FMI), weight status) and covariates. Linear and logistic regression models were fit, including adjustment for DED and moderate-to-vigorous physical activity. Results showed that baseline DED was associated with change in WC (*β* for 1kJ/g difference: 0·71; 95% CI 0·26, 1·17), particularly in boys (1·26; 95% CI 0·41, 2·16 *v*. girls: 0·26; 95% CI −0·34, 0·87), but not with %BF, FMI or weight status. In contrast, baseline SED, MPA or VPA were not associated with any of the outcomes. Change in DED was negatively associated with FMI (*β* for 1kJ/g increase: −0·86; 95% CI −1·59, −0·12) and %BF (−0·86; 95% CI −1·25, −0·11) but not WC (−0·27; 95% CI −1·02, 0·48). Change in SED, MPA and VPA did not predict adiposity at follow-up. In conclusion, activity intensity was not prospectively associated with adiposity, whereas the directions of associations with DED were inconsistent. To inform public health efforts, future studies should continue to analyse longitudinal data to further understand the independent role of different energy-balance behaviours in changes in adiposity in early adolescence.

The development of obesity is known to be largely the result of energy imbalance, in which energy intake (EI) exceeds energy expenditure over a prolonged period^(^ ^1^ ^)^. The most recent UK prevalence data show that when leaving primary school, one in three children are either overweight or obese^(^ ^2^ ^)^. A similar prevalence has been reported in the USA^(^ ^3^ ^)^, indicating a need for public health action. A recent meta-analysis of interventions for preventing obesity in children showed that those targeting changes in both physical activity and dietary behaviour were marginally more effective in reducing weight gain than those only targeting one^(^ ^4^ ^)^, although through what behavioural mechanism this is achieved is unclear. Although this observation is consistent with abundant cross-sectional evidence, the precise role of activity and diet behaviours in adiposity development remains unclear^(^ ^5^ ^)^. Moreover, the limited available evidence exhibits a number of limitations including the use of imprecise measures of activity, insufficient consideration of the impact of changes in the exposures and limited adjustment for confounding^(^ ^6^ ^–^ ^8^ ^)^.

Recent reviews highlight the lack of evidence of an association between objectively measured physical activity^(^ ^8^ ^,^ ^9^ ^)^ or sedentary time^(^ ^6^ ^)^ and changes in adiposity in young people. It has been suggested that the lack of association between total physical activity and subsequent changes in adiposity may mask associations with subcomponents of physical activity^(^ ^9^ ^)^. Indeed, a cross-sectional study suggests that activity of vigorous intensity is more strongly associated with adiposity outcomes than moderate activity or total physical activity^(^ ^10^ ^)^. One recent prospective study additionally showed that, independent of diet quality and activity of lower intensity, participation in objectively measured vigorous activity at baseline was associated with a beneficial change in several health outcomes over a 2-year follow-up period^(^ ^11^ ^)^. However, this study only considered a limited number of confounders (age, sex and diet quality assessed with 24-h recall) and did not assess the impact of change in behavioural exposures. Moreover, despite suggestions that time spent sedentary may be an important independent risk factor for children’s health^(^ ^12^ ^,^ ^13^ ^)^, recent prospective studies have failed to confirm this association^(^ ^14^ ^,^ ^15^ ^)^.

In contrast, the limited available prospective epidemiological evidence consistently indicates energy-dense diets as a contributing factor to EI and excess weight gain in childhood^(^ ^7^ ^,^ ^16^ ^,^ ^17^ ^)^. Dietary energy density (DED) refers to the amount of energy consumed given the weight of food reportedly consumed, and therefore likely to be less prone to under-reporting than the absolute measure of EI. Prospectively, DED has been associated with subsequent changes in adiposity in childhood, independent of EI^(^ ^18^ ^)^. However, no study has investigated changes in DED and adiposity, and few prospective studies adjust for the potential contribution of physical activity^(^ ^19^ ^)^. It therefore remains unclear whether and how physical activity and dietary factors influence weight development in children.

In light of the limitations of the current evidence base, we aimed to quantify the independent association between (change in) activity intensity (e.g. time spent in sedentary, moderate or vigorous activity) and DED and change in adiposity over a 4-year follow-up period in a population-based sample of young British adolescents. We tested the following complementary hypotheses: (1) behaviour at baseline predicts change in adiposity; and (2) change in behaviour predicts adiposity at follow-up.

## Methods

### Study overview

The SPEEDY study (Sport, Physical activity and Eating behavior: Environmental Determinants in Young people) is a population-based longitudinal cohort study set in the county of Norfolk, UK^(^ ^20^ ^)^. Ethics approval for the whole study was obtained from the University of East Anglia Research Ethics Committee; parental informed consent and student assent were obtained at all measurement occasions. The analyses presented here used data from baseline and 4-year follow-up.

### Study procedures

The complete details of SPEEDY participant recruitment and study procedures for the baseline^(^ ^20^ ^)^ and follow-up^(^ ^21^ ^)^ data collection have been detailed elsewhere. In brief, at baseline, primary schools in Norfolk were purposively sampled to achieve urban and rural heterogeneity. In total, 157 schools were approached (out of 227 eligible schools with ≥12 Year 5 children), of which ninety-two were recruited and participated in the study. Invitation packs were handed out to all Year 5 children (*n* 3619, aged 9–10 years). A total of 2064 children provided valid consent and were measured at baseline (57% response rate). Baseline data collection took place during school visits between April and July 2007. At 4-year follow-up, all the participants with valid baseline home addresses (*n* 1964) were sent an invitation pack. Researchers additionally gave presentations at secondary schools attended by at least five original SPEEDY participants to encourage participation. Consent forms (signed by both parents and participants) were returned to the study office by mail. Follow-up data collection took place at schools (or at home if more convenient) between April and August 2011.

### Data collection procedures

At both time points, researchers visited schools to take physical measurements, administer self-report questionnaires, fit accelerometers and hand out 4-d food diaries; participants returned the accelerometers and the diaries to school 1 week later.

#### Anthropometry assessment

Trained research assistants used standardised protocols to measure participants’ height and weight. Height was measured to the nearest millimetre (Leicester height measure; Chasmors Ltd). A non-segmental bio-impedance scale was used to measure weight (to the nearest 0·1kg) and impedance in light clothing (type TBF-300A; Tanita). Height and weight measures were used to calculate BMI (kg/m^2^). Weight status was derived using International Obesity Task Force sex- and age-dependent cut-off points^(^ ^22^ ^)^. Previously validated and published procedures^(^ ^23^ ^)^ using eight equations were used to calculate fat mass and body fat percentage^(^ ^24^ ^–^ ^31^ ^)^. Fat mass index (FMI) was presented as fat mass/height^2^ (kg/m^2^). Waist circumference (WC) was measured twice to the nearest millimetre at the midpoint between the lower costal margin and the level of the anterior superior iliac crests, using a Seca 200 measuring tape (Seca). A third measurement was taken if a discrepancy of ≥3cm was observed and an average was calculated. All scales were calibrated before and halfway through data collection. Quality assurance was established by assessing inter-observer variability of height and WC before and after data collection, which was found to be acceptable (ranging from 0·1 to 0·4cm for height and 0·7 to 1·3cm for WC).

#### Physical activity assessment

Physical activity was objectively assessed using an Actigraph accelerometer (model GT1M; Actigraph). The Actigraph has been shown to have validity in assessing physical activity among children during free-living conditions^(^ ^32^ ^,^ ^33^ ^)^, although it cannot accurately assess water-based activities and cycling. All monitors were calibrated before first use and regularly throughout the study. The monitor was set to record the vertical acceleration at 5-s epochs. Participants were asked to wear the monitors during waking hours for 7d and to remove them while undertaking water-based activities.

Data were analysed using a batch processing programme (MAHUffe, available at: www.mrc-epid.cam.ac.uk/research/resources) to remove any data recorded after 23.00 hours and before 06.00 hours. Periods of 10min or more that had continuous zero activity counts and any days with <500min of recording were excluded^(^ ^34^ ^,^ ^35^ ^)^. Participants were included if they provided ≥3d of valid data at both time points.

Physical activity at baseline and at follow-up for each individual was summarised as average daily time spent sedentary (SED) and in moderate (MPA), vigorous (VPA) and moderate-to-vigorous physical activity (MVPA). Thresholds for defining activity intensities were as follows: SED<100counts per min (cpm), MPA 2000–3999 and VPA≥4000cpm; MVPA was defined as ≥2000cpm^(^ ^36^ ^,^ ^37^ ^)^ and scaled to 5-s epochs.

#### Dietary assessment and processing

Dietary intake was assessed using a 4-d food diary – a method previously validated in 9–10-year-old children^(^ ^38^ ^)^ and applied in young adolescents^(^ ^39^ ^)^. Assessment days were consecutive, concurrent with physical activity measurement and included 2 weekdays and 2 weekend days. Participants recorded, with assistance of their parents or care givers, all foods and drinks consumed, and estimated the portion size of each item. Participants practiced completion during the measurement session by reporting on the foods and drinks consumed that day, on which they were given feedback to improve the level of detail provided. The weights of the portions were then approximated using published values for children^(^ ^40^ ^–^ ^42^ ^)^. Diaries were coded at Medical Research Council Human Nutrition Research (Cambridge, UK) using Diet-In Nutrients-Out^(^ ^43^ ^)^, which uses continually updated British food composition data.

Daily DED (kJ/g) was estimated as total EI from food (kJ) relative to total grams of food consumed, excluding beverages and supplements, which has been shown to provide a more valid estimate of DED^(^ ^44^ ^)^. Beverages included the following: tea, coffee, other hot beverages, water (drink), milk (drink), milkshakes, dairy smoothies (excluding fruit smoothies), yogurt drinks, diluted juices/squash, fruit juices and all carbonated and non-carbonated soft drinks. Items added to beverages, including sugar, dried beverage powders and milk were excluded from DED calculations.

Dietary misreporting can bias diet–disease associations, and dietary under-reporting is common among adolescents^(^ ^45^ ^)^. We quantified dietary misreporting as the ratio of daily EI relative:estimated energy requirements (EER, estimated as total energy expenditure plus energy required for growth)^(^ ^46^ ^)^. As cut-offs to identify under- and over-reporters may be subject to error^(^ ^47^ ^)^ and as the average EI:EER at baseline was 0·67 (sd 0·16), indicating that under-reporting was very common in this cohort, EI:EER was included as a continuous variable^(^ ^48^ ^)^.

#### Covariates

Inclusion of covariates focused on accounting for alternative important determinants of adiposity in youth, such as parental weight status, birth weight, sleep duration and puberty status^(^ ^49^ ^–^ ^51^ ^)^. Data on maternal BMI (calculated from self-reported height and weight), parent-reported child’s birth weight and age when the main care giver left full-time education (self-reported and categorised as ≤16, 16–18, >18 years) were collected via a self-administered parental baseline questionnaire. At the age of 14 years, parents reported on five puberty signs from which a sum-score (0–5) was derived – three general (growth spurt, body hair growth, spots/acne) and two sex-specific (boys: deepening voice, facial hair; girls: breast growth, menarche) scores. Average sleep duration at baseline was derived from the child’s self-reported bedtime and wake time during school days and weekends, with the mean recorded as average sleep duration in hours^(^ ^10^ ^)^. Age and sex were self-reported during the measurement sessions; average accelerometer wear time (min/d) was derived from the processed accelerometer data; and DED from drinks and dietary misreporting were established using the procedures described above.

### Statistical analyses

All the analyses were performed using STATA version 13. Baseline age, sex, BMI *Z* score and the care giver’s age when leaving full-time education were compared between those included and excluded from the analysis, with *P* values for these comparisons calculated from logistic regression with exclusion/inclusion as the binary outcome, and robust standard errors to allow for school-level clustering. We then modelled four adiposity variables (WC, FMI, %body fat (%BF) and weight status (overweight/obese *v*. normal weight)) and four exposure variables (time spent in SED, MPA and VPA and DED). For each outcome/exposure combination, a series of linear (continuous outcome) or logistic (binary outcome) regression models were fit, with robust standard errors to account for clustering within schools. Two complementary analytical approaches were used for each outcome/exposure combination:

(A) Regression of outcome on baseline exposure, adjusted for baseline value of outcome (e.g. baseline SED with change in FMI).

(B) Regression of outcome on change in exposure (e.g. change in SED with follow-up FMI).

First, a basic model with adjustment for age and sex was run for all analyses (model 1). Subsequent models also included socio-economic status, birth weight, maternal BMI, puberty status at follow-up and sleep (model 2); reciprocal adjustment for MVPA and DED (model 3); and reciprocal adjustment for MVPA and SED (activity-related exposures only, model 4). All models with activity-related exposures were additionally adjusted for baseline/change in accelerometer wear time; DED models were additionally adjusted for baseline/change in DED from drinks^(^ ^44^ ^)^ and baseline under-reporting. Models with WC as the outcome were adjusted for height at baseline. To account for the potential association between baseline behaviour and change in behaviour, all models including change in behaviour were additionally adjusted for the baseline value of that behaviour. Differences in exposure/outcome associations by sex and baseline weight status were investigated by including an interaction term between the moderating variable and the main exposure of interest in model 3. Effects in subgroups were estimated when the *P* value for the test of interaction was <0·1. To assess the influence of missing data, we ran sensitivity analyses restricting the sample to those with full data only. Differences from the main results were minimal and did not affect conclusions (data not reported).

## Results

Of 2064 baseline participants, 1964 (95·2) had valid contact details, of which 480 were re-recruited at the 4-year follow-up (24·4%); 424 (88%) returned an Actigraph monitor, with 367 (76·4% of follow-up sample) providing valid data at both baseline and follow-up on physical activity and/or DED. Those included in the analyses (*n* 367) did not differ from those excluded (*n* 1697) with respect to sex and baseline age and BMI *Z* scores. However, on average, they did come from more highly educated families (age parent left full-time education (≤16, 16–18, >18 years): included: 30·0, 36·5, 21·5% *v*. excluded: 44·9, 27·9, 17·4%; *P*=0·027). Table 1 shows baseline characteristics and mean change in exposure, outcome and confounding variables, stratified by sex.

**Table 1:** Baseline characteristics and observed change in exposures and outcomes in the analytical sample (Mean values and standard deviations)

	Boys (*n* 175)		Girls (*n* 192)	
	Mean/%	sd/*n*	Mean/%	sd/*n*
Demographic data				
Age (years)	9·7	0·5	9·8	0·4
Maternal BMI (kg/m^2^)	24·8	4·9	25·0	4·7
Sleep time (h)	10·4	0·9	10·7	0·7
Age the care giver left full-time education (%)				
≤16 years	33·7	59	43·8	84
16–18 years	38·9	68	34·4	66
>18 years	22·9	40	20·3	39
Anthropometry				
Waist (cm)				
Baseline	64·3	7·6	62·9	7·8
4-year change	8·2	5·2	8·8	5·6
FMI (kg/m^2^)				
Baseline	7·9	3·8	9·4	4·9
4-year change	4·4	4·2	7·8	4·5
%body fat				
Baseline	21·0	5·6	24·9	6·1
4-year change	−0·5	3·9	4·5	4·1
%overweight/obese (baseline)	18·5	32	23·4	45
Diet diary-derived data				
DED (kJ/g)				
Baseline	7·6	1·3	7·9	1·3
4-year change	0·6	1·5	0·3	1·6
Accelerometer-derived data				
SED (min)				
Baseline	453·4	51·3	468·0	52·8
4-year change	48·0	72·6	41·1	65·8
MPA (min)				
Baseline	53·6	13·9	45·2	11·8
4-year change	−9·9	17·8	−1·6	16·6
VPA (min)				
Baseline	30·9	15·6	22·0	11·1
4-year change	−7·4	17·0	−6·4	15·4
Wear time (min)				
Baseline	722·8	55·5	715·7	61·2
4-year follow-up	716·0	70·8	703·8	61·2

FMI, fat mass index; DED, dietary energy density; SED, sedentary physical activity; MPA, moderate physical activity; VPA, vigorous physical activity.

Table 2 shows the results of linear regression analyses using baseline exposure and change in adiposity (analytical approach A). The activity-related exposures were not associated with change in any of the outcomes. Baseline DED was, however, positively associated with change in WC independent of baseline MVPA. Only one significant interaction was identified, suggesting that the main effect of baseline DED on change in WC differed by sex. Subsequent subgroup analyses indicated that the positive effect was statistically significant in boys but not in girls (*β* for boys: 1·28; 95% CI 0·41, 2·16 *v*. girls: 0·26; 95% CI −0·34, 0·87).

**Table 2:** Associations between baseline behaviour and change in adiposity (analytical approach A)* (*β*-Coefficients and 95% confidence intervals)

	WC		FMI		%body fat	
Behaviours	*β*	95% CI	*β*	95% CI	*β*	95% CI
SED (min/d)†						
Model 1‡	−0·14	−0·32, 0·04	−0·02	−0·17, 0·13	0·02	−0·09, 0·14
Model 2§	−0·12	−0·31, 0·07	−0·01	−0·16, 0·14	0·01	−0·11, 0·13
Model 3||	−0·14	−0·31, 0·03	−0·06	−0·21, 0·08	−0·04	−0·13, 0·05
Model 4¶	−0·10	−0·43, 0·23	−0·11	−0·40, 0·19	−0·05	−0·25, 0·14
MPA (min/d)†						
Model 1‡	0·49	0·00, 0·99	0·05	−0·27, 0·38	−0·05	−0·35, 0·25
Model 2§	0·54	−0·01, 1·10	0·06	−0·25, 0·38	−0·03	−0·32, 0·27
Model 3||	0·57	−0·01, 1·15	0·20	−0·17, 0·58	0·11	−0·19, 0·40
Model 4¶	0·54	−0·45, 1·53	0·11	−0·56, 0·78	0·003	−0·59, 0·60
VPA (min/d)†						
Model 1‡	0·22	−0·24, 0·69	−0·05	−0·40, 0·29	−0·09	−0·39, 0·20
Model 2§	0·06	−0·37, 0·49	−0·18	−0·44, 0·08	−0·15	−0·43, 0·13
Model 3||	0·20	−0·22, 0·62	−0·01	−0·25, 0·23	0·05	−0·19, 0·28
Model 4¶	−0·09	−0·71, 0·52	−0·20	−0·65, 0·26	−0·04	−0·39, 0·31
DED**						
Model 1‡	0·67	0·25, 1·09	0·31	0·07, 0·55	0·27	0·03, 0·52
Model 2§	0·69	0·23, 1·15	0·22	−0·08, 0·52	0·18	−0·14, 0·50
Model 3||	0·71	0·26, 1·17	0·22	−0·08, 0·52	0·18	−0·14, 0·50

WC, waist circumference; FMI, fat mass index; SED, sedentary physical activity; MPA, moderate physical activity; VPA, vigorous physical activity; DED, dietary energy density; PA, physical activity; MPVA, moderate-to-vigorous physical activity.

* *β* Represents the estimated difference in mean change in outcome per 10min (PA/SED) or 1kJ/g (DED) increase in exposure. *n* included in the analyses varies between 245 and 336, depending on exposure and outcome.

† Additionally adjusted accelerometer-registered time.

‡ Model 1: age, sex.

§ Model 2: model 1+socio-economic status, birth weight, maternal BMI, puberty status at follow-up, sleep duration; model with WC as outcome also adjusted for height.

|| Model 3: model 2+baseline DED for PA exposures and baseline MVPA for DED.

¶ Model 4: model 3+objectively measured sedentary time (for MPA and VPA exposures) or MVPA (for SED exposure).

** DED models additionally adjusted for energy intake (kJ) from drinks and baseline under-reporting.

Table 3 presents the results of linear regression analyses of change in exposure and adiposity at follow-up (analytical approach B). For the activity-related exposures, the estimated associations were generally not in the expected direction, although the 95% CI were wide and compatible with no association. In contrast, after adjustment for confounders (model 2), change in DED was significantly, and negatively, associated with FMI and %BF at follow-up, but not with WC. Further adjustment for MVPA made very little difference to the estimates of association. Only one significant interaction was identified, suggesting that the effect of changes in VPA on FMI at follow-up differed by obesity status. However, although the estimated *β*-coefficients were in opposite directions in normal weight and overweight/obese children, both 95% CI were compatible with no association (*β* for normal weight: 0·46; 95% CI −0·18, 1·11, overweight/obese: −1·13; 95% CI −3·92, 0·17).

**Table 3:** Associations between change in behaviour and adiposity at follow-up (analytical approach B)* (*β*-Coefficients and 95% confidence intervals)

	WC		FMI		%body fat	
	*β*	95% CI	*β*	95% CI	*β*	95% CI
SED (min/d)†						
Model 1‡	−0·03	−0·30, 0·23	−0·04	−0·24, 0·15	−0·07	−0·23, 0·10
Model 2§	−0·01	−0·24, 0·23	−0·06	−0·24, 0·12	−0·05	−0·22, 0·11
Model 3||	−0·002	−0·27, 0·27	−0·09	−0·30, 0·12	−0·09	−0·27, 0·08
Model 4¶	0·24	−0·25, 0·72	0·17	−0·23, 0·57	0·09	−0·23, 0·40
MPA (min/d)†						
Model 1‡	0·37	−0·31, 1·05	0·19	−0·34, 0·71	0·25	−0·23, 0·72
Model 2§	0·16	−0·50, 0·81	0·08	−0·46, 0·62	0·12	−0·38, 0·63
Model 3||	0·10	−0·63, 0·83	0·02	−0·58, 0·62	0·03	−0·47, 0·54
Model 4¶	0·03	−1·04, 1·11	−0·10	−1·00, 0·81	−0·23	−0·91, 0·44
VPA (min/d)†						
Model 1‡	0·35	−0·33, 1·02	0·24	−0·33, 0·80	0·26	−0·24, 0·76
Model 2§	0·36	−0·43, 1·14	0·19	−0·45, 0·84	0·19	−0·37, 0·76
Model 3||	0·30	−0·58, 1·18	0·39	−0·30, 1·09	0·40	−0·22, 1·02
Model 4¶	0·48	−0·49, 1·45	0·51	−0·26, 1·28	0·47	−0·22, 1·15
DED**						
Model 1‡	−0·43	−1·20, 0·34	−0·63	−1·23, −0·02	−0·52	−1·03, −0·01
Model 2§	−0·26	−1·01, 0·49	−0·83	−1·56, −0·10	−0·66	−1·22, −0·09
Model 3||	−0·27	−1·02, 0·48	−0·86	−1·59, −0·12	−0·68	−1·25, −0·11

WC, waist circumference; FMI, fat mass index; SED, sedentary physical activity; MPA, moderate physical activity; VPA, vigorous physical activity; DED, dietary energy density; PA, physical activity; MPVA, moderate-to-vigorous physical activity.

* *β* Represents the estimated difference in mean level of outcome per 10min (PA/SED) or 1kJ/g (DED) increase in exposure. *n* included in the analyses varies between 245 and 335, depending on exposure and outcome.

† Additionally adjusted for change in accelerometer-registered time.

‡ Model 1: age, sex, baseline value of exposure.

§ Model 2: model 1+socio-economic status, birth weight, maternal BMI, puberty status at follow-up, sleep duration; model with WC as outcome also includes height.

|| Model 3: model 2+change in DED for PA/SED exposures and change in MVPA for DED.

¶ Model 4: model 3+objectively measured change in sedentary time (for MPA and VPA exposures) or change in MVPA (for SED exposure).

** DED models additionally adjusted for change energy intake (kJ) from drinks and baseline under-reporting.

The results of logistic regression analyses using weight status as the outcome are presented in Table 4. There was very little evidence to suggest that either baseline behaviour or change in behaviour was associated with being overweight/obese at follow-up. Increasing SED was associated with lower odds of overweight/obesity at follow-up; however, this association became non-significant after adjustment for change in MVPA. A significant interaction suggested that the influence of baseline SED on change in obesity status differed by sex. However, although the estimated OR were in opposite directions in boys and girls, both 95% CI were compatible with no association (OR for girls: 0·97; 95% CI 0·82, 1·14, and boys: 1·25; 95% CI 0·92, 1·72).

**Table 4:** Associations between baseline behaviour/change in behaviour and odds of being overweight/obese at follow-up* (Odds ratios and 95% confidence intervals)

	Baseline behaviour†		Change in behaviour	
	OR	95% CI	OR	95% CI
SED (min/d)‡				
Model 1§	1·07	0·97, 1·18	0·94	0·88, 1·01
Model 2||	1·05	0·96, 1·15	0·92	0·84, 1·00
Model 3¶	1·01	0·91, 1·13	0·90	0·81, 0·99
Model 4**	1·02	0·85, 1·22	0·95	0·78, 1·14
MPA (min/d)‡				
Model 1§	0·81	0·58, 1·11	1·11	0·94, 1·31
Model 2||	0·89	0·65, 1·21	1·09	0·89, 1·33
Model 3¶	1·00	0·69, 1·46	1·11	0·86, 1·41
Model 4**	1·13	0·54, 2·37	0·92	0·65, 1·29
VPA (min/d)‡				
Model 1§	0·73	0·53, 1·00	1·13	0·93, 1·37
Model 2||	0·78	0·53, 1·16	1·13	0·84, 1·51
Model 3¶	0·94	0·63, 1·41	1·17	0·87, 1·57
Model 4**	0·95	0·57, 1·59	1·11	0·77, 1·60
DED††				
Model 1§	1·18	0·89, 1·57	1·02	0·83, 1·26
Model 2||	1·03	0·73, 1·45	0·94	0·71, 1·23
Model 3**	1·01	0·72, 1·42	0·94	0·72, 1·22

SED, sedentary physical activity; MPA, moderate physical activity; VPA, vigorous physical activity; DED, dietary energy density; PA, physical activity; MVPA, moderate-to-vigorous physical activity.

* OR of being overweight/obese per 10min (PA) or 1kJ/g (DED) increase in exposure. *n* included in the analyses varies between 245 and 334, depending on exposure and outcome.

† Additional adjustment for BMI *Z* score at baseline.

‡ Additionally adjusted accelerometer-registered time.

§ Model 1: age, sex.

|| Model 2: model 1+socio-economic status, birth weight, maternal BMI, puberty status at follow-up, sleep duration.

¶ Model 3: model 2+baseline DED for PA exposures and baseline MVPA for DED.

** Model 4: model 3+objectively measured sedentary time (for MPA and VPA exposures) or MVPA (for SED exposure).

†† DED models additionally adjusted for energy intake (kJ) from drinks and baseline under-reporting.

## Discussion

This study shows that, in early adolescence, objectively measured activity intensity is not prospectively associated with adiposity markers, whereas the direction of results for DED was inconsistent. We are therefore unable to draw robust conclusions about the importance of energy-balance behaviours for obesity prevention. Strengths of the study include the longitudinal design, the relatively large population-based sample with objectively measured physical activity, detailed food diary data and objective anthropometry measures at two time points in a challenging age group. However, a key limitation is the cohort attrition, impacting on the wider generalisability of the results, particularly as those included in the present analyses on average came from higher educated families than the original cohort.

As suggested by previous review evidence^(^ ^7^ ^,^ ^16^ ^)^, DED was associated with changes in indicators of adiposity. The results showed that a higher DED at baseline was associated with a greater 4-year increase in WC, but not FMI, %BF or weight status. A higher baseline DED of 1kJ/g was associated with a 0·71cm greater increase in WC. This association was independent of a number of potential confounders previously under-explored in the literature, such as birth weight, maternal BMI and sleep duration. Models were additionally adjusted for EI from drinks and dietary under-reporting; further adjustment for objectively measured physical activity did not attenuate the observed association. In addition to addressing the more commonly studied predictors of change in adiposity, we also investigated the association between changes in DED and adiposity indicators at follow-up. To our knowledge, no study has explored this in an adolescent population. Unexpectedly, the results showed that an increase in DED was associated with lower FMI and lower %BF at follow-up, whereas no association was observed with WC. There are several hypotheses for the observed negative association. First, DED at baseline might have reached a ceiling level for children with high baseline adiposity, whereas increases in DED were feasible for smaller children at initially lower levels. Baseline weight status did not, however, moderate the association, raising doubt about this hypothesis. Second, changes in DED might have been due to changes in energy expenditure, and therefore changes in energy requirements. However, adjustment for minutes of MVPA did not attenuate the association. Third, reporting bias in dietary assessment is known to be weight dependent^(^ ^45^ ^)^. Analyses were adjusted for baseline dietary under-reporting, resulting in minimal attenuation (data not shown). The likely impact of potential changes in reporting bias due to increased weight is therefore considered minimal. Whatever the reason, the overall mixed results observed in this study prevent drawing robust conclusions about the longitudinal association between DED and adiposity in early adolescence and warrant further exploration in sufficiently large samples with multiple robust measures of exposure, outcome and potentially confounding variables.

The role of physical activity in weight gain has been a long-standing issue of discussion. Despite an abundance of evidence for a cross-sectional association, particularly for activity of higher intensity, overall, the results of longitudinal and interventional studies have been mixed^(^ ^6^ ^,^ ^9^ ^,^ ^52^ ^)^. In the present study, the results were consistent between activity-related exposures and generally counter-intuitive, but not statistically significant. Although one might argue that this may be due to a small sample size, the size of the effect estimates additionally indicates that the observed associations are unlikely to be of clinical relevance. Results from the logistic regression model using weight status at follow-up as the outcome showed that a 10-min increase in SED was associated with 10% lower odds of being overweight or obese at follow-up, after controlling for known confounders and DED. However, this association was attenuated and became non-significant after adjustment for MVPA. A recent review of longitudinal evidence on the association between objectively measured sedentary time and adiposity^(^ ^6^ ^)^ identified only three studies, two of which reported a null association with the remaining study showing a positive association. Longitudinal evidence that sedentary time, or indeed specific sedentary behaviours (in particular television viewing)^(^ ^53^ ^)^, is positively associated with adiposity therefore remains weak at best. Although reductions in specific sedentary behaviours may be beneficial, calls to reduce overall sedentary time to reduce obesity at a population level may therefore be premature.

The common approach to modelling the prospective association between health behaviours and outcome is to regress change in outcome on baseline behaviour (as presented here in Table 2). The data presented here would have enabled development of a change model (where change in behaviour is related to change in adiposity). However, as in cross-sectional analyses, identification of the direction of association is not possible in these models, and this analytical strategy was therefore not pursued. The clear temporal sequences of the models presented in this study were hypothesised to provide a clearer indication of the potential direction of association, and therefore a step closer to the identification of any causal associations. However, the lack of associations for the activity-related models and the conflicting results for the DED models prevent this.

Reverse causality in the obesogenic behaviour–adiposity relationship needs to be considered in light of the results presented in this study. Recent data in children suggest that adiposity predicts lower levels of physical activity^(^ ^54^ ^)^ and higher amounts of sedentary time^(^ ^55^ ^)^. This has also been suggested in adults^(^ ^56^ ^,^ ^57^ ^)^. Although plausible, there are also methodological reasons for this observation. Both outcome and exposure variables are measured with error, but behavioural variables are generally measured with greater error than anthropometric data. Random measurement error in the exposure variable leads to an attenuation of the association to zero. However, random measurement error in the outcome variable increases the standard errors, and therefore impacts the precision, but not the estimate of effect^(^ ^58^ ^)^. Efforts to improve the validity and reduce measurement error of the measures of behaviour are therefore crucial to improve our understanding of the causality of the association under investigation.

### Conclusions

This is one of the first studies to investigate the independent prospective association between activity intensity, DED and measures of adiposity in a population-based sample of young adolescents. No evidence was shown for a prospective association between SED, MPA or VPA and adiposity indicators, whereas the evidence for a prospective association between DED and adiposity was mixed and varied by outcome and analytical approach applied. On the basis of these results, no robust conclusions can be drawn on the associations of the impact of activity intensity and DED with weight gain. Future work should focus on analysing longitudinal data using diverse approaches in sufficiently large samples, with valid measures of the behaviours and sufficient follow-up. In addition, the role of reverse causality and the potential prospective impact of activity intensity and dietary behaviours on non-adiposity outcomes (such as mental well-being, academic performance and bone health) should be considered more consistently to inform public health policy. From a public health perspective, promoting increased physical activity and healthy eating, and decreased consumption of energy-dense foods, remains an important public health target, even if such changes may have minimal impact on adiposity.
